# Recurrence of Pancreatic Neuroendocrine Tumors and Survival Predicted by Ki67

**DOI:** 10.1245/s10434-018-6518-2

**Published:** 2018-05-22

**Authors:** C. G. Genç, M. Falconi, S. Partelli, F. Muffatti, S. van Eeden, C. Doglioni, H. J. Klümpen, C. H. J. van Eijck, E. J. M. Nieveen van Dijkum

**Affiliations:** 1 Department of Surgery, Academic Medical Center, Amsterdam, The Netherlands; 2 Pancreatic Surgery Unit, Pancreas Translational and Research Institute, Scientific Institute, San Raffaele Hospital, University Vita e Salute, Milan, Italy; 3 Department of Pathology, Academic Medical Center, Amsterdam, The Netherlands; 4 Department of Pathology, Scientific Institute, San Raffaele Hospital, University Vita e Salute, Milan, Italy; 5 Department of Medical Oncology, Academic Medical Center, Amsterdam, The Netherlands; 6 Cancer Center Amsterdam, Amsterdam, The Netherlands; 7 Department of Surgery, Erasmus Medical Center, Rotterdam, The Netherlands

## Abstract

**Background:**

Despite evidence of different malignant potentials, postoperative follow-up assessment is similar for G1 and G2 pancreatic neuroendocrine tumors (panNETs) and adjuvant treatment currently is not indicated. This study investigated the role of Ki67 with regard to recurrence and survival after curative resection of panNET.

**Methods:**

Patients with resected non-functioning panNET diagnosed between 1992 and 2016 from three institutions were retrospectively analyzed. Patients who had G1 or G2 tumor without distant metastases or hereditary syndromes were included in the study. The patients were re-categorized into Ki67 0–5 and Ki67 6–20%. Cox regression analysis with log-rank testing for recurrence and survival was performed.

**Results:**

The study enrolled 241 patients (86%) with Ki67 0–5% and 39 patients (14%) with Ki67 6–20%. Recurrence was seen in 34 patients (14%) with Ki67 0–5% after a median period of 34 months and in 16 patients (41%) with Ki67 6–20% after a median period of 16 months (*p* < 0.001). The 5-year recurrence-free and 10-year disease-specific survival periods were respectively 90 and 91% for Ki67 0–5% and respectively 55 and 26% for Ki67 6–20% (*p* < 0.001). The overall survival period after recurrence was 44.9 months, which was comparable between the two groups (*p* = 0.283). In addition to a Ki67 rate higher than 5%, tumor larger than 4 cm and lymph node metastases were independently associated with recurrence.

**Conclusions:**

Patients at high risk for recurrence after curative resection of G1 or G2 panNET can be identified by a Ki67 rate higher than 5%. These patients should be more closely monitored postoperatively to detect recurrence early and might benefit from adjuvant treatment. A clear postoperative follow-up regimen is proposed.

One of the concerns for patients with pancreatic neuroendocrine tumors (panNETs) is the accurate prediction of clinical outcome. Tumor stage and grade have proved to be useful in estimating disease course and have been confirmed repeatedly in valuable studies.^1^^–^6 Despite this, follow-up assessment is the same for all patients who have undergone curative resection of panNET. Neither surveillance protocols nor adjuvant treatment options based on expected recurrence rates are available, although the recurrence rate is reported to be 17% after resection of well-differentiated panNET, with considerable consequences for survival.^7^

The 2010 tumor grade classification of the World Health Organization (WHO) divides panNET into three prognostic groups based on the proliferation index assessed through the expression of the nuclear antigen Ki67, with Ki67 < 3% classified as low-grade panNET (G1), Ki67 3–20% classified as intermediate-grade panNET (G2), and Ki67 > 20% classified as high-grade neuroendocrine carcinoma (NEC) (G3).^8^^–^11

Multiple studies have shown a good correlation between the Ki67 index and tumor size, angioinvasion, and biologic behavior of neuroendocrine tumors.^12^^–^14 However, heterogeneity of panNET is increasingly described, and the wide range of the Ki67 distribution in the grading systems is under debate.^15^^–^17 Therefore, WHO proposed an updated classification system for panNET this year, in which high-grade tumors with Ki67 > 20% are subdivided into well-differentiated G3 NET and poorly differentiated G3 NEC.^18^ Although clear upper or lower limits for G3 NET and G3 NEC are not provided, differences in genetic basis and the course of disease are suggested.^19^^–^21 Similar assumptions also are apparent for tumors with Ki67 < 20%. A Ki67 cutoff of 10% is used to select patients suitable for liver transplantation according to the Milan criteria, comparable with the inclusion criteria of the Clarinet study and of many oncologists generally when choosing a systemic treatment.^22^^,^^23^

For non-metastasized patients, the latest European Neuroendocrine Tumor Society (ENETS) guidelines also discriminate between “low-G2” and “high-G2” panNET without providing cutoff values, suggesting different treatment responses within this patient population.^1^ Furthermore, several studies describe a higher discriminating capacity when G1 and G2 panNET are divided by a Ki67 cutoff of 5% instead of 3% to predict disease progression.^5^^,^^24^^,^^25^

After curative surgery of panNET, follow-up assessment is focused on early detection of recurrence. The use of the Ki67 proliferation index to guide postoperative management has not been described previously.^7^^,^^26^^,^^27^ Based on the capacity of Ki67 to predict disease outcome in general, it is likely that the proliferation index of surgically treated panNET could also be predictive in estimating the risk for the development of recurrence. Therefore, we hypothesized that panNET with Ki67 < 20% indicates a heterogeneous group of tumors with a different postoperative disease course and aimed to investigate the role of Ki67 in predicting recurrence and survival after curative resection.

## Methods

The study enrolled patients who underwent a curative resection of a non-functioning panNET with Ki67 < 20% between 1992 and 2016 from the following three academic centers: The Academic Medical Center Amsterdam and The Erasmus University Rotterdam in the Netherlands (both ENETS Centers of Excellence) and the Ospedale San Raffaele in Milan, Italy. The data for 211 patients (75%) also have been presented in a previous study of this group.^7^

All the patients were free of distant metastatic disease at diagnosis and not associated with a genetic predisposition for the development of panNET. Pathology reports were reviewed for the diagnosis of panNET, and patients were included in the study if panNET was histologically proven. All patients with (unresectable) locally advanced or distant metastatic disease, successfully treated or not, were excluded from the study.

The functional status of the tumors was based on the clinical presentation of symptoms associated with hormonal overproduction. The Ki67 proliferation index was retrieved from pathology reports. Tumor tissue of patients with a diagnosis before 2010 or with pathology reports containing insufficient information on the Ki67 index (*n* = 24) were reassessed with an emphasis on Ki67 by experienced pathologists.

For all the patients, visual assessment (“eyeballing”) was used to assess Ki67, and histologic grade was based on the WHO classification of 2010.^28^ Classification according to the Royal College of Pathologists was used to assess resection margins.^29^ Depending on the tumor location, pancreaticoduodenectomy, distal pancreatectomy, or total pancreatectomy was performed. Central pancreatectomy or enucleation was performed for patients with a small panNET far enough from the pancreatic duct. Lymphadenectomy was not routinely performed with enucleation.

**Fig. 1:**
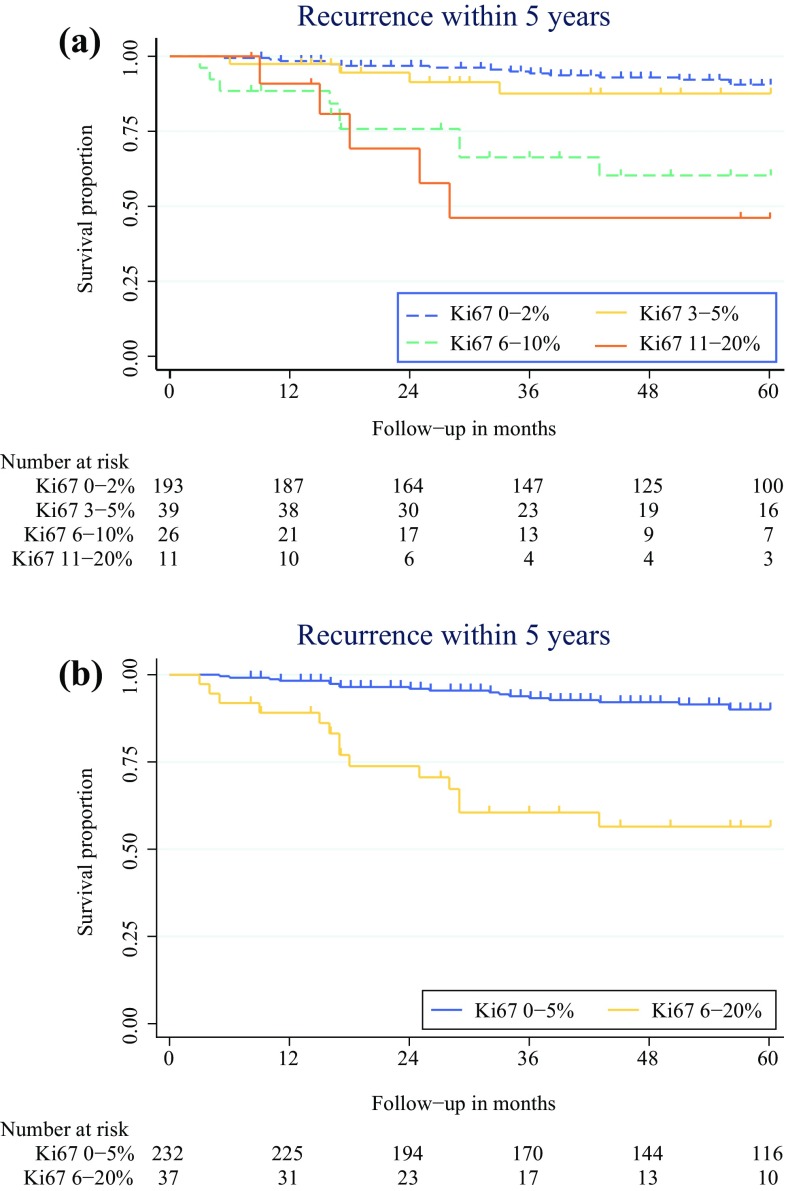
Recurrence within 5 years after curative resection. **A** Patients categorized into four groups based on Ki67. **B** Patients categorized in two groups based on Ki67

The patients were categorized into groups based on the Ki67 proliferation index of the tumor. Because pathologists frequently did not report an exact number to indicate Ki67, but rather provided a range for the proliferation rate, groups were initially defined by the most commonly used cutoffs provided in the pathology reports as follows: G1 (Ki67 0–2%), low G2 (Ki67 3–5%), mid-G2 (Ki67 6–10%), and high G2 (Ki67 11–20%). Because early Kaplan–Meier analysis (Fig. 1a) showed similar results for patients with G1 and low G2, as well as for patients with mid-G2 and high G2, and because the cutoff of 5% also was supported by Cox proportional hazard regression (Table 1), the patients were re-categorized into two groups: Ki67 0–5 and Ki67 6–20% (Fig. 1b).

**Table 1:** Predictors for recurrence within 5 years (*n* = 280)

	Univariate Cox regression			Multivariable Cox regression		
	HR	95% CI	*p* value	HR	95% CI	*p* value
Male sex	0.99	0.49–2.00	0.976			
Age (years)						
< 40	Ref	Ref	Ref			
41–50	0.72	0.18–2.88	0.639			
51–60	0.86	0.23–3.26	0.826			
61–70	1.06	0.30–3.74	0.923			
> 70	0.20	0.02–1.95	0.167			
Tumor location						
Head	Ref	Ref	Ref			
Body	0.86	0.36–2.05	0.738			
Tail	0.85	0.37–1.97	0.704			
Tumor size (mm)						
< 20	Ref	Ref	Ref			
21–40	2.45	0.84–7.16	0.102			
> 41	6.13	2.24–16.75	<0.001	2.27	1.10–4.72	0.027
R1 resection	1.72	0.71–4.19	0.233			
WHO tumor grade	0.24	0.12–0.47	<0.001	–	–	–
Ki67 (%)						
0–2	Ref	Ref	Ref			
3–5	1.99	0.72–5.52	0.188			
6–10	5.88	2.46–14.05	<0.001			
11–20	7.68	2.52–23.42	<0.001			
Ki67 > 5%	5.54	2.68–11.43	<0.001	5.21	1.47–18.4	0.010
Positive lymph nodes	4.95	2.32–10.58	<0.001	3.36	1.48–7.61	0.004
Perineural invasion	3.17	1.41–7.17	0.005	–	–	–
Vascular invasion	3.09	1.50–6.37	0.002	–	–	–

*HR* hazard ratio, *CI* confidence interval, *WHO* World Health Organization

Follow-up assessment after resection consisted of physical exams, laboratory tests, and radiologic imaging. The frequency of hospital visits was at least every 6 months for the first 2 years and yearly thereafter. Follow-up time was defined as the time to the last known date the patient was alive or the time until death. Recurrence was defined as local recurrence in the pancreas, a new location in lymph nodes, or the development of distant metastases. All recurrences were identified through radiologic imaging.

Statistical analyses were performed using IBM SPSS Statistics 23 (IBM Corp., Armonk, NY). On the basis of the distribution, the data were described using mean and standard deviation (SD) or using median and interquartile range (IQR). For categorical data, the number and proportion (%) were displayed. Differences between patient and tumor characteristics were investigated using a Chi-square statistic for categorical values and a Mann–Whitney *U* test for numeric values.

Kaplan–Meier survival analyses with log-rank testing were performed to investigate recurrence-free and disease-specific survival. To identify variables associated with recurrence within 5 years after surgery, Cox proportional hazard regression analyses were performed. Receiver-operating-characteristic (ROC) analysis with area-under-curve (AUC) determination was performed to investigate the diagnostic ability with regard to recurrence and disease-specific survival. The results were presented with the hazard ratio (HR) and the 95% confidence interval (CI). The discriminative ability of the model was examined by calculating the Harrel c-statistic with 95%.^30^ Moreover, we examined the discrimination of the WHO grade model and compared the c-statistics of the two models using a *z* test. The net reclassification improvement (NRI) analysis was used to quantify how well our new proposed model reclassified subjects compared with the current WHO grading classification.^31^^,^^32^

## Results

This study analyzed 280 patients. Patient and tumor characteristics are presented in Table 2. Left pancreatectomy was performed for 136 patients (49%), pancreaticoduodenectomy for 80 patients (29%), enucleation for 45 patients (16%), central pancreatectomy for 13 patients (5%) and total pancreatectomy for 5 patients (2%).

**Table 2:** Tumor and patient characteristics (*n* = 280)

	*n* (%)
Male:female	136:144
Median age: years (IQR)	59 (48.8–66)
Median follow-up: months (IQR)	62 (36–84)
Tumor location	
Head	105 (38)
Body	81 (29)
Tail	94 (34)
Mean Ki67 (%)	2.8 ± 3.7
0–2	199 (71)
3–5	42 (15)
6–10	28 (10)
11–20	11 (4)
Median tumor size: mm (IQR)	25 (15–40)
< 20	113 (40)
21–40	100 (36)
> 40	67 (24)
R0:R1	240:39
Lymph node metastases	65 (23)
Missing (%)	12
Perineural invasion	34 (13)
Missing (%)	9
Vascular invasion	65 (25)
Missing (%)	5
Recurrence	49 (18)
Local	12 (25)
Regional	4 (8)
Distant	26 (53)
Unknown location	7 (14)
Median size: mm (IQR)	40 (25–59)
Mean Ki67 (%)	4.8 ± 5.4
G2^a^	23/49 (47)
R1 resection	13/49 (27)
Lymph node metastases	27/49 (55)
Perineural invasion	13/49 (27)
Vascular invasion	23/49 (47)
Median time to recurrence: months (IQR)	31.7 (10.5–47)
Median survival after recurrence: months (IQR)	44.9 (16–68.3)
> 30-day mortality	25 (9)
Disease-related deaths	14 (5)

*IQR* interquartile range

^a^According to the 2010 World Health Organization (WHO) classification^8^

Tumors with Ki67 0–5% were seen in 241 patients, whereas 39 patients had a panNET with Ki67 6–20%. The patients with Ki67 6–20% more frequently had lymph node metastases (53 vs 22%; *p* = 0.0002), perineural invasion (28 vs 11%; *p* = 0.0129), vascular invasion (51 vs 20%; *p* < 0.0001), and R1 resection (36 vs 12%; *p* = 0.0438) than the patients with Ki67 0–5%.

### Recurrence and Survival

Recurrence was experienced by 49 patients (18%), and the majority (53%) of these recurrences were located in distant organs. The patients with recurrence more often had tumors in the pancreatic head (45 vs 36%; *p* = 0.0174), tumors larger than 2 cm (86 vs 54%; *p* < 0.0001), WHO 2010 grade 2 tumors (47 vs 25% G1; *p* = 0.0033), R1 resection (26 vs 11%; *p *= 0.0126), lymph node metastases (60 vs 19%; *p* < 0.0001), perineural invasion (30 vs 10%; *p* = 0.0004), and vascular invasion (49 vs 19%; *p* = 0.0342) than the patients without recurrence.

Of the 241 patients with Ki67 0–5%, 34 (14%) had a recurrence. Local recurrence in the pancreas of 12 patients was observed and recurrence in the regional lymph nodes of 2 patients. Distant metastases developed in 18 patients. Of the 39 patients with Ki67 6–20%, 16 (41%) had a recurrence, with 1 found locally in the pancreas, 2 found in regional lymph nodes, and 8 found as distant metastases. Kaplan–Meier analysis showed significantly less recurrence within 5 years after surgery for the patients with Ki67 0–5% than for the patients with Ki67 6–20% (*p* < 0.001; Fig. 1b). The 5-year recurrence-free survival rate was 90% for the patients with Ki67 0–5 and 55% for the patients with Ki67 6–20%. Overall, the median time to recurrence (TTR) was 31.7 months (IQR 10.5–47 months): 34 months (IQR 16–59 months) for the patients with Ki67 0–5% and 16 months (IQR 4.25–23.25 months) for the patients with Ki67 6–20% (*p* = 0.005).

**Fig. 2:**
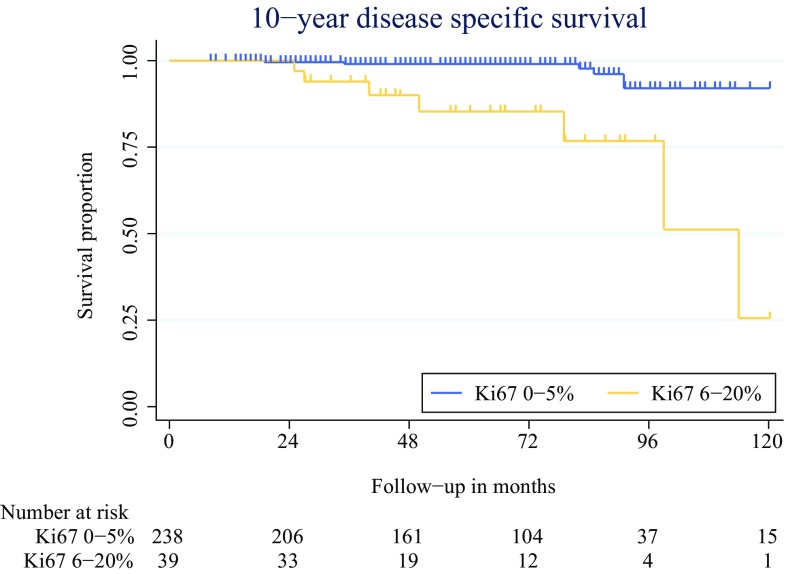
The 10-year disease-specific survival times for patients with Ki67 0–5 and Ki67 6–20%

The median survival time was 63 months for the patients with Ki67 0–5% tumors and 45 months for the patients with Ki67 6–20% tumors (*p* = 0.017). The 10-year disease-specific survival was 91% for the patients with Ki67 0–5% tumors and 26% for the patients with Ki67 6–20% tumors (*p* < 0.001, Fig. 2). The median survival time after recurrence was 44.9 months (IQR 16–68.3 months), which was statistically comparable between the two groups (*p* = 0.283).

The ROC analysis for Ki67 showed an AUC of 0.683 for the prediction of recurrence within 5 years. The highest sensitivity and specificity were reached at a Ki67 cutoff value of 5%, with a sensitivity of 37% and a specificity of 87%. An AUC of 0.737 was found for 10-year disease-specific survival.

The discriminative ability of this Ki67 model showed a Harrel c-statistic of 0.672 (95% CI 0.591–0.753). The discrimination of the WHO grading with regard to predicting recurrence was comparable, with a c-statistic of 0.681 (95% CI 0.602–0.760). This was not statistically significant (*p* = 0.781).

### Net Reclassification Improvement Analysis

Table 3 presents the results of the NRI analysis. The additive NRI of the proposed Ki67 cutoff value was 0.866, indicating that the new cutoff value had good additive value for the WHO grading classification. The absolute NRI was 10%, indicating that 10% of patients were correctly reclassified in our proposed model based on their risk for the development of recurrence within 5 years. This effect can best be attributed to the reclassification of patients with a low risk for the development of recurrence.

**Table 3:** Reclassification of patients with and without recurrence

WHO grading model	New proposed Ki67 cutoff		
	Ki67 0–5%	Ki67 6–20%	Total
Patients with recurrence (*n* = 49, 17.5%)			
Grade 1	26	0	26
Grade 2	7	16	23
Total	33	16	49
Patients without recurrence (*n* = 231, 82.5%)			
Grade 1	173	0	173
Grade 2	35	23	58
Total	208	23	231

Net reclassification of patients with recurrence: 0 − 7 = − 7. Net reclassification of patients without recurrence: 35 − 0 = 35. Additive net reclassification improvement (NRI) analysis: ([− 7/49] × 100) + ([35/231] × 100) = 0.866. Absolute NRI analysis: ([− 7 + 35]/280) × 100 = 10%

*WHO* World Health Organization

### Cox Proportional Hazard Analysis

The factors related to recurrence within 5 years after surgery from the univariable Cox regression analyses were tumor size greater than 4 cm, WHO tumor grade, Ki67 > 5%, lymph node metastases, and perineural and vascular invasion. The independent predictors for recurrence were tumor size greater than 4 cm (HR 2.5; 95% CI 1.14–5.40), Ki67 > 5% (HR 3.0; 95% CI 1.34–6.81), and lymph node metastases (HR 3.3; 95% CI 1.40–7.70) (Table 1). Tumors larger than 4 cm were seen in 67 patients, 21 (31%) of whom experienced a recurrence. The absolute NRI of Ki67 compared with size was 6.5%. Lymph node metastases were present in 65 patients, 27 of whom experienced recurrence (42%). The absolute NRI of Ki67 compared with lymph node metastases was 5%.

The 10-year disease-specific survival was associated with Ki67 > 5% and perineural invasion in the univariate analysis, but only Ki67 > 5% was independently associated with 10-year disease specific survival in the multivariable Cox regression analysis (HR 6.5; 95% CI 1.93–21.79; *p* = 0.003).

## Discussion

We propose a novel categorization of low- and intermediate-grade panNET based on the Ki67 index to predict recurrence after curative resection. Tumors with Ki67 6–20% have a threefold higher risk for the development of recurrence within 5 years and show significantly shorter survival than tumors with Ki67 ≤ 5%. With this cutoff value, a reliable method for stratifying patients into groups of high and low risk for recurrence after surgery is presented.

In a previous study, we presented a scoring system to identify high-risk patients through three predictors for recurrence.^7^ The current study contributes to strengthening of this scoring system. When the criteria for grade 2 tumors are modified for tumors with Ki67 > 5%, it will be possible to identify high-risk patients more accurately. The recurrence score showed a sensitivity of 91% and a specificity of 62% and is expected to increase with this revision. Furthermore, patients with Ki67 3–5% (15% of our cohort) will be downgraded by this modification, limiting unnecessary treatment or monitoring. External validation of the scoring system currently is being performed and will include this new Ki67 distribution as well.

Postoperative follow-up assessment of patients with panNET typically consists of hospital visits combined with laboratory tests and/or radiologic or nuclear imaging. A clear guideline for postoperative management such as the frequency of hospital visits, the method for diagnostic testing, or the duration of follow-up assessment has not been recommended to date. Combining the presented results with preexistent literature, we propose a postoperative surveillance protocol based on the risk of recurrence for patients who have non-metastasized panNET with K67 < 20% (Table 4). This scheme comprises yearly consultations with imaging for all patients and additional half-yearly consultations with clinical assessments and laboratory tests (chromogranin A) for high risk-patients. Based on clinical findings and laboratory results, additional imaging may be obtained.

**Table 4:** Surveillance protocol after curative resection of pancreatic neuroendocrine tumor (panNET) with Ki76 < 20%

	Yearly follow-up	Additional follow-up	Frequency	Duration (years)
Low-risk patients^a^	Clinical assessment imaging^b^	–	Yearly	≥ 5
High-risk patients^a^	Clinical assessment imaging^b^	Clinical assessment laboratory tests^c^	Every 6 months	10

^a^Risk stratification either through the newly proposed Ki67 distribution, or more accurately through the modified version of the recurrence score by Genç et al.^7^

^b^Alternating between anatomic and nuclear methods

^c^Chromogranin A

Ideally, imaging is alternated between radiologic and somatostatin receptor imaging to achieve the highest accuracy. Findings have shown that gallium-based nuclear imaging has the highest sensitivity and specificity for the detection of panNET and is therefore the preferred nuclear imaging method.^33^^–^36 Radiologic imaging with either contrast-enhanced computed tomography (CT) or (diffusion weighted) magnetic resonance imaging (MRI) is advised.^37^^–^39 Based on the median time to recurrence, a follow-up period of 10 years is encouraged because late recurrences have been described.^7^ The interval between assessments can be increased if the disease is stable after 5 years, especially for low-risk patients.

Due to the retrospective nature of this study, it was not possible to assess exact Ki67 rates for each patient. It is questionable, however, whether exact rates for each tumor will be more meaningful in determining postoperative prognosis. At this writing, exact Ki67 values have limited clinical relevance because the choice for treatment is often determined by tumor grade or smaller ranges of Ki67. Furthermore, the proliferation index of a tumor may have different prognostic significance in different stages of disease or treatment. This is already evident, for example, in determination of systemic treatment options for patients with disseminated disease. A Ki67 cutoff of 10% often is used by oncologists, confirming heterogeneity in malignant potential within one WHO grading group. The treatment of localized nonfunctioning tumors smaller than 2 cm might also be influenced by different Ki67 cutoffs, in which the choice for surgical versus conservative treatment may change for G2 tumors with higher or lower Ki67 values. In addition, assessing the exact amount of Ki67-positive cells, either manually on printed images or determined through computer software, also can create a false sense of accuracy because each method for counting positive cells is associated with an error margin. Likewise, differences in practice can lead to intra- and interobserver variability. Therefore, it might be both more reliable and more feasible to agree on smaller ranges of Ki67 (e.g., < 5, 5–10, 15–20%) rather than exact values, with stratification of patients into their risk for the development of recurrence.

The current results must be seen in light of their limitations. Data were evaluated retrospectively, and pathology reports were not standardized at the time of treatment. Furthermore, the treatment of recurrence was not taken into account when survival was analyzed. Because survival after recurrence was comparable between Ki67 0–5 and Ki67 6–20% tumors, we expect the treatment of these patients to be similar. Nevertheless, results might be biased, and survival after recurrence might show treatment results rather than the effect of recurrence itself. In addition, these results could be interpreted with the assumption that early detection and treatment of recurrence will result in survival benefit. However, no studies support this theory, and prospective clinical trials are necessary to confirm these hypotheses.

At this writing, the clinical relevance of this study may be limited except for de-escalation of follow-up regimens for Ki67 0–5% patients and intensification of follow-up regimens for patients with Ki67 6–20%. Adjuvant therapy to prevent recurrence in the future could be a possibility. However, the vicious circle of nonexistent data, together with the difficulty of obtaining prospective studies for this purpose, forms an obstacle to the development of such treatments. To overcome these issues, a consensus study has been initiated among European panNET experts to discuss possibilities for investigating the role of adjuvant treatment for high-risk patients. The results of this consensus will be published shortly. The current study might bring us one step closer to achieving this necessary research by clarifying the selection of patients who should be eligible for adjuvant treatment.

In conclusion, this study is the largest study to describe the use of the Ki67 proliferation index to estimate postoperative recurrence. These results contribute to the assumption of tumor heterogeneity among patients with a Ki67 < 20%. Future studies should focus on determining Ki67 rates, preferably in prospective trials, to propose a further alteration of the grading system for well-differentiated panNET.

## References

[1] Falconi M, Eriksson B, Kaltsas G (2016). ENETS Consensus guidelines update for the management of patients with functional pancreatic neuroendocrine tumors and non-functional pancreatic neuroendocrine tumors. Neuroendocrinology..

[2] Fischer L, Kleeff J, Esposito I (2008). Clinical outcome and long-term survival in 118 consecutive patients with neuroendocrine tumours of the pancreas. Br J Surg..

[3] La Rosa S, Klersy C, Uccella S (2009). Improved histologic and clinicopathologic criteria for prognostic evaluation of pancreatic endocrine tumors. Hum Pathol..

[4] Pape UF, Jann H, Muller-Nordhorn J (2008). Prognostic relevance of a novel TNM classification system for upper gastroenteropancreatic neuroendocrine tumors. Cancer..

[5] Scarpa A, Mantovani W, Capelli P (2010). Pancreatic endocrine tumors: improved TNM staging and histopathological grading permit a clinically efficient prognostic stratification of patients. Mod Pathol..

[6] Pezzilli R, Partelli S, Cannizzaro R (2016). Ki-67 prognostic and therapeutic decision driven marker for pancreatic neuroendocrine neoplasms (PNENs): a systematic review. Adv Med Sci..

[7] Genc CG, Jilesen AP, Partelli S (2018). A new scoring system to predict recurrent disease in grade 1 and 2 nonfunctional pancreatic neuroendocrine tumors. Ann Surg..

[8] Klimstra DS, Modlin IR, Coppola D, Lloyd RV, Suster S (2010). The pathologic classification of neuroendocrine tumors: a review of nomenclature, grading, and staging systems. Pancreas..

[9] Endl E, Gerdes J (2000). The Ki-67 protein: fascinating forms and an unknown function. Exp Cell Res..

[10] Farrell JM, Pang JC, Kim GE, Tabatabai ZL (2014). Pancreatic neuroendocrine tumors: accurate grading with Ki-67 index on fine-needle aspiration specimens using the WHO 2010/ENETS criteria. Cancer Cytopathol..

[11] Delle Fave G, Kwekkeboom DJ, Van Cutsem E (2012). ENETS consensus guidelines for the management of patients with gastroduodenal neoplasms. Neuroendocrinology..

[12] Rindi G, Kloppel G, Couvelard A (2007). TNM staging of midgut and hindgut (neuro) endocrine tumors: a consensus proposal including a grading system. Virchows Arch..

[13] Rindi G, Kloppel G, Alhman H (2006). TNM staging of foregut (neuro)endocrine tumors: a consensus proposal including a grading system. Virchows Arch..

[14] Genc CG, Klumpen HJ, van Oijen MGH, van Eijck CHJ, Nieveen van Dijkum EJM (2018). A nationwide population-based study on the survival of patients with pancreatic neuroendocrine tumors in The Netherlands. World J Surg..

[15] Reid MD, Balci S, Saka B, Adsay NV (2014). Neuroendocrine tumors of the pancreas: current concepts and controversies. Endocr Pathol..

[16] Ricci C, Casadei R, Taffurelli G (2014). WHO 2010 classification of pancreatic endocrine tumors. is the new always better than the old?. Pancreatology..

[17] Ricci C, Casadei R, Taffurelli G (2016). Validation of the 2010 WHO classification and a new prognostic proposal: a single-centre retrospective study of well-differentiated pancreatic neuroendocrine tumours. Pancreatology..

[18] Lloyd RV, Osamura RY, Klöppel G, Rosai J. *WHO Classification of Tumous of Endocrine Organs,* 4th ed., vol 10. Lyon, France: International Agency for Research on Cancer (IARC); 2017.

[19] Velayoudom-Cephise FL, Duvillard P, Foucan L (2013). Are G3 ENETS neuroendocrine neoplasms heterogeneous?. Endocr Relat Cancer..

[20] Tanaka H, Matsusaki S, Baba Y (2015). Neuroendocrine tumor G3: a pancreatic well-differentiated neuroendocrine tumor with a high proliferative rate. Clin J Gastroenterol..

[21] Garcia-Carbonero R, Sorbye H, Baudin E (2016). ENETS Consensus guidelines for high-grade gastroenteropancreatic neuroendocrine tumors and neuroendocrine carcinomas. Neuroendocrinology..

[22] Mazzaferro V, Pulvirenti A, Coppa J (2007). Neuroendocrine tumors metastatic to the liver: how to select patients for liver transplantation. J Hepatol..

[23] Caplin ME, Pavel M, Cwikla JB (2014). Lanreotide in metastatic enteropancreatic neuroendocrine tumors. N Engl J Med..

[24] Pelosi G, Bresaola E, Bogina G (1996). Endocrine tumors of the pancreas: Ki-67 immunoreactivity on paraffin sections is an independent predictor for malignancy: a comparative study with proliferating-cell nuclear antigen and progesterone receptor protein immunostaining, mitotic index, and other clinicopathologic variables. Hum Pathol..

[25] Rindi G, Falconi M, Klersy C (2012). TNM staging of neoplasms of the endocrine pancreas: results from a large international cohort study. J Natl Cancer Inst..

[26] Ballian N, Loeffler AG, Rajamanickam V, Norstedt PA, Weber SM, Cho CS (2009). A simplified prognostic system for resected pancreatic neuroendocrine neoplasms. HPB Oxford..

[27] Birnbaum DJ, Gaujoux S, Cherif R (2014). Sporadic nonfunctioning pancreatic neuroendocrine tumors: prognostic significance of incidental diagnosis. Surgery..

[28] Bosman FT, Carneiro F, Hruban RH, Theise ND. *WHO Classification of Tumors,* 4th ed., vol 3. Lyon, France: International Agency for Research on Cancer (IARC); 2010.

[29] Stephenson TJ, Cross SS, Chetty R. Standards and datasets for reporting cancers: dataset for neuroendocrine tumors of the gastrointestinal tract including pancreas, 3rd ed. *R Coll Pathol.* 2013;12–13. http://ukeps.com/docs/gimds.pdf.

[30] Newson R (2006). Confidence intervals for rank statistics: percentile slopes, differences, and ratios. Stata J..

[31] Alba AC, Agoritsas T, Walsh M (2017). Discrimination and calibration of clinical prediction models: users’ guides to the medical literature. JAMA..

[32] Leening MJ, Vedder MM, Witteman JC, Pencina MJ, Steyerberg EW (2014). Net reclassification improvement: computation, interpretation, and controversies: a literature review and clinician’s guide. Ann Intern Med..

[33] Sadowski SM, Neychev V, Millo C (2016). Prospective study of 68 Ga-DOTATATE positron emission tomography/computed tomography for detecting gastro-entero-pancreatic neuroendocrine tumors and unknown primary sites. J Clin Oncol..

[34] Mojtahedi A, Thamake S, Tworowska I, Ranganathan D, Delpassand ES (2014). The value of (68)Ga-DOTATATE PET/CT in diagnosis and management of neuroendocrine tumors compared to current FDA-approved imaging modalities: a review of literature. Am J Nucl Med Mol Imaging..

[35] Haidar M, Shamseddine A, Panagiotidis E (2017). The role of 68 Ga-DOTA-NOC PET/CT in evaluating neuroendocrine tumors: real-world experience from two large neuroendocrine tumor centers. Nucl Med Commun..

[36] Panagiotidis E, Alshammari A, Michopoulou S (2017). Comparison of the impact of 68 Ga-DOTATATE and 18F-FDG PET/CT on clinical management in patients with neuroendocrine tumors. J Nucl Med..

[37] Farchione A, Rufini V, Brizi MG (2016). Evaluation of the added value of diffusion-weighted imaging to conventional magnetic resonance imaging in pancreatic neuroendocrine tumors and comparison with 68 Ga-DOTANOC positron emission tomography/computed tomography. Pancreas..

[38] Semelka RC, Custodio CM, Cem Balci N, Woosley JT (2000). Neuroendocrine tumors of the pancreas: spectrum of appearances on MRI. J Magn Reson Imaging..

[39] Foti G, Boninsegna L, Falconi M, Mucelli RP (2013). Preoperative assessment of nonfunctioning pancreatic endocrine tumours: role of MDCT and MRI. Radiol Med..

